# The Microbial Diversity in Relation to Postharvest Quality and Decay: Organic vs. Conventional Pear Fruit

**DOI:** 10.3390/foods12101980

**Published:** 2023-05-12

**Authors:** Qi Gao, Yang Zhang, Congcong Gao, Huimin Li, Yudou Cheng, Xun Qian, Lishu Zhang, Jinyu Liu, Solabomi Olaitan Ogunyemi, Junfeng Guan

**Affiliations:** 1 Institute of Biotechnology and Food Science, Hebei Academy of Agricultural and Forestry Sciences, Shijiazhuang 050051, China; 2 Key Laboratory of Plant Genetic Engineering Center of Hebei Province, Shijiazhuang 050051, China; 3 School of Landscape and Ecological Engineering, Hebei Engineering University, Handan 056021, China; 4 Cangzhou Academy of Agricultural and Forestry Sciences, Cangzhou 061001, China; 5 State Key Laboratory of Rice Biology, Ministry of Agriculture Key Lab of Molecular Biology of Crop Pathogens and Insects, Institute of Biotechnology, Zhejiang University, Hangzhou 310013, China

**Keywords:** pear, management systems, microbial communities, decay, storage

## Abstract

(1) Background: Organic food produced in environmentally friendly farming systems has become increasingly popular. (2) Methods: We used a DNA metabarcoding approach to investigate the differences in the microbial community between organic and conventional ‘Huangguan’ pear fruit; and (3) Results: Compared to a conventional orchard, the fruit firmness in the organic orchard had significantly lowered after 30 days of shelf-life storage at 25 °C, and the soluble solids content (SSC), titratable acid (TA), and decay index were higher. There were differences in the microbial diversity between organic and conventional orchards pears. After 30 days of storage, *Fusarium* and *Starmerella* became the main epiphytic fungi in organic fruits, while *Meyerozyma* was dominant in conventional fruits. *Gluconobacter*, *Acetobacter*, and *Komagataeibacter* were dominant epiphytic bacteria on pears from both organic and conventional orchards after a 30-day storage period. *Bacteroides*, *Muribaculaceae*, and *Nesterenkonia* were the main endophytic bacteria throughout storage. There was a negative correlation between fruit firmness and decay index. Moreover, the abundance of *Acetobacter* and *Starmerella* were positively correlated with fruit firmness, while *Muribaculaceae* was negatively correlated, implying that these three microorganisms may be associated with the postharvest decay of organic fruit; (4) Conclusions: The difference in postharvest quality and decay in organic and conventional fruits could potentially be attributed to the variation in the microbial community during storage.

## 1. Introduction

Organic food refers to food that is grown, harvested, stored and processed without the use of artificial synthetic substances, such as chemical fertilizers, pesticides and hormones. Organic food has become increasingly popular in the last decade, which has promoted the global market growth while improving food quality [1]. Organic farming methods help to preserve the natural taste and nutritional value of food [2]. Organic food producers, processors, and consumers prioritize environmental safety while highlighting the sustainable development of humanity, nature, and society [3]. For these reasons, organic management for orchards has become widespread in fruit production.

Orchard managements have significant impacts on the nutritional properties of fruits [4,5]. There is a difference in quality of fruits and vegetables between organic and conventional orchards [6], and the antioxidant defense system was significantly improved in organic orchards [7,8]. However, it was found that apples from two management systems resulted in a similar total soluble solids content, juice pH, titratable acidity, and color indexes [9]. Similar results were also reported for plums from two orchards under organic and conventional managements [10]. Therefore, the effect of organic management on fruit quality varies among different fruits. The absence of pesticides and fertilizers may stimulate organic plants to protect themselves and produce more secondary metabolites [11]. These compounds can enhance the fruit’s aroma by adding sweeter and fruitier scents, which are the accumulation of flavor substances in organic fruit. However, and probably due to the absence of chemical fungicide application, it was found that diseases in organic orchards were more severe than in conventional orchards. For example, apple scab (*Venturia inaequalis*) in apples [12] and leaf spots (*Septoria lycopersici* and *Xanthomonas vesicatoria*) in tomatoes [9].

Numerous reports have confirmed that changes in the microbial community structure play an important role in the occurrence of postharvest fruit decay [13,14,15,16,17,18,19]. During long-term storage, the abundance of pathogens on the fruit surface increases, exacerbating the risk of postharvest fruit spoilage [20]. Despite the presence of diverse microorganisms, most microorganisms in the fruit microbial community are not pathogenic. However, the function of these microorganisms in maintaining fruit quality, physiological characteristics, and disease resistance during postharvest storage is not yet clear.

In agroecosystems, it has been shown that orchard management practices affect the composition and structure of the plant microbiome [21]. Cultivation methods also have a significant impact on the nutritional properties and aroma of the fruit [22]. The effect of different management systems of orchards on pear fruit microbiome communities is currently unknown. However, the application of metagenomics has provided a fundamental breakthrough in the description, comparison, and discovery of new microbial communities [23].

‘Huangguan’ pear (*Pyrus bretschneideri* Rehd cv. Huangguan) is a widely planted cultivar in China, but fruit decay caused by pathogens limits its postharvest shelf life [24]. In this study, high-throughput sequencing techniques were used to investigate changes in fungal and bacterial communities in epiphytic and endophytic microorganisms on fruit from organic and conventional orchards, and the relationship between pear quality, postharvest decay, and microbial composition.

## 2. Materials and Methods

### 2.1. Fruit Sample Collection

‘Huangguan’ pears were harvested at the commercial maturity stage on 20 August 2021 [25] from two orchards with organic or conventional management systems located in Haixing County, Hebei Province, China (117.41228 °E, 38.01518 °N). To eliminate the possibility of soil and climate variation, as well as the risk of pesticide contamination in the organic orchard, the two orchards (ten years old) were located 2 km apart. Both orchards were under routine management, the conventional orchards used pesticides for pest management, while the organic orchard did not use pesticides or fertilizers. Fruits were bagged with three-layer paper bags on 30 days after fruit setting. Fruit weighed 0.25 ± 0.06 kg per fruit at harvest. Fruits were transported within 4 h to the laboratory immediately after harvesting and afterwards were stored at 25 °C for 0, 15, and 30 days under the relative humidity of 80 ± 2%. In this experiment, a total of 120 fruit were harvested from organic orchards and conventional orchards, respectively.

### 2.2. Microbial Sample Collection

Epiphytic microorganisms were obtained by wiping the surface of fruit with a sterile cotton swab, which was then placed in a 1.5 mL centrifuge tube (45 fruit, 5 replicates with 3 fruit each for 3 time points). For endophytic microorganisms sampling, the fruit was ultrasonically treated at 40 Hz for 15 min using an ultrasonic machine (KS-500DE, Kunshan Jielimei Ultrasonic Instrument Co., LTD., Kunshan, China), followed by immersing in 10% sodium hypochlorite solution for 1 min, which was then immersed in sterile water and rinsed three times. Then, about 2 mm thick and 1 cm^2^ in size of the fruit was peeled off and placed in a 1.5 mL centrifuge tube. Samples were frozen in liquid nitrogen and stored at −80 °C for further use. Five replicates were used for each treatment with three fruit per replicate. Treatments (i.e., organic or conventional; storage times) were labeled as follows for epiphytic microorganisms: Or0-ep, Or15-ep and Or30-ep/Co0-ep, Co15-ep and Co30-ep. Similarly, samples for the determination of endophytic microorganism were labeled as follows: Or0-en, Or15-en and Or30-en/Co0-en, Co15d_en and Co30-en.

### 2.3. Determination of Fruit Quality and Decay

Fruit firmness, soluble solids contents (SSC), titratable acidity (TA), decay rate, and decay index were determined according to previous reports [26]. For firmness, fruit were peeled approximately 2 mm thick and 1 cm^2^ in size at the equator and measured with a handheld firmness tester (GY-4, Tuopu, China). SSC was measured using a PAL-1 handheld digital brix meter (ATGAO, Tokyo, Japan). TA was determined by acid-base titration method. Fifteen fruit (5 replicates with 3 fruit each) were used for the destructive quality measurements (firmness, SSC, and TA) per treatment (organic/conventional) and per storage time.

The decay rate was calculated as the ratio of the number of decay fruit to the total number of fruit. Three replicates with 10 fruit (30 fruit in total) were used in each treatment. Fruit decay is classified into four grades: 0 = 0% surface is decayed; 1 = <25% surface is decayed; 2 = <50% surface is decayed; and 3 = >75% surface is decayed. There were three replicates, each containing 10 fruit. The decay index was calculated as follows: ∑ (number of decay fruit at each level × representative value of each level)/(total number of fruit surveyed × representative value of the highest level).

### 2.4. DNA Extraction and Illumina Sequencing

Primers ITS1 (CTTGGTCATTTAGAGGAAGTAA) and ITS4 (GCTGCGTTCTTCATCGATGC) were used to amplify the fungal ITS gene, and 799F (AACMGGATTAGATACCCKG) and 1193R (ACGTCATCCCCACCTTCC) were used to amplify the bacterial 16S gene [26]. PCR products were sequenced on the Illumina MiSeq/NovaSeq platform at Personal Biotechnology in Shanghai, China. After removal of barcode sequences by sequence analysis, sequence denoising was performed according to the DADA2 analysis process in QIIME2 [27].

### 2.5. Statistical Analysis

Significant differences between treatments were tested by *t*-test. The taxonomic composition, alpha diversity, and random forests were analyzed by QIIME2 after adjusting all samples to the same sequencing depth. For the alpha diversity, the Shannon index was used to assess the richness and evenness of species within a sample, with larger values indicating higher richness among species. Beta diversity (PCoA) was analyzed by ImageGP using the Bray–Curtis dissimilarity, which is generally sensitive to observing differences between groups, resulting in lower sample size [28]. Pearson correlation analysis was performed and plotted by using the genescloud tools (https://www.genescloud.cn/ (accessed on 16 September 2022)). Graphs of fruit quality and decay were generated by using GraphPad Prism 9 software (GraphPad Inc., San Diego, CA, USA).

## 3. Results

### 3.1. Effect of Different Pest Management Systems on Fruit Quality and Decay during Storage

**Figure 1:**
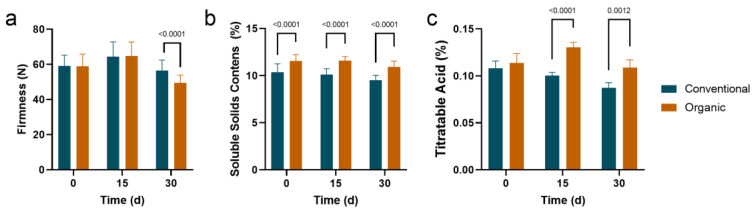
Changes in the firmness (**a**), soluble solids content (**b**), and titratable acidity (**c**) of ‘Huangguan’ pears from organic and conventional orchards. Each treatment was replicated 5 times, and each replicate contained 3 fruit. The between-group differences were tested using *t*-tests, and the *p*-values between groups with significant differences are displayed.

As shown in Figure 1a, after 30 days of storage, firmness was significantly lower in organic fruit than in conventional pear fruit. The SSC of organic fruit was significantly higher than that of conventional fruit throughout the storage (Figure 1b). Throughout storage, organic fruit had higher TA values than conventional fruit, but no significant difference was observed in TA between organic and conventional fruits at the beginning of storage. However, after 15 and 30 days of storage, the TA values in organic pear fruit were significantly higher than those in conventional pear fruit (Figure 1c).

**Figure 2:**
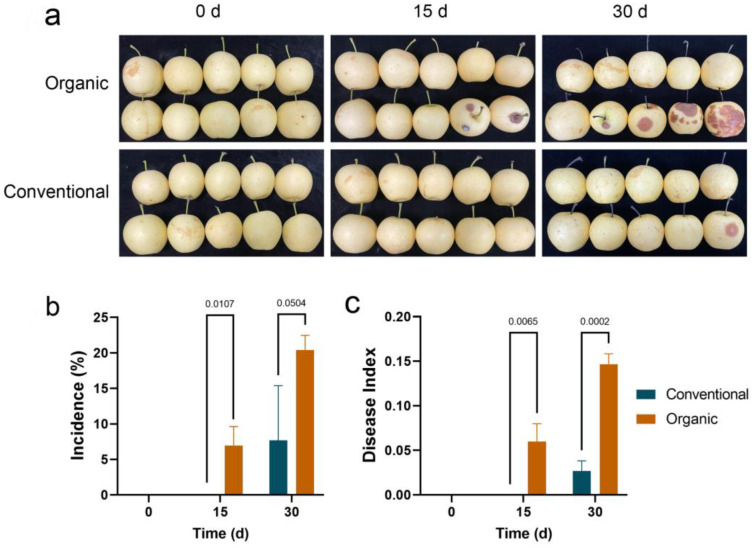
The postharvest decay of ‘Huangguan’ pears from organic and conventional orchards during storage. (**a**): Representative fruit pictures. (**b**): Incidence. (**c**): Decay index. There were three replicates, each containing 10 fruit.

As shown in Figure 2, in organic fruit, decay appeared after 15 days of storage with incidence reaching 20% after 30 days of storage, while in conventional fruit, decay appeared after 30 days and was significantly lower than the organic fruit. Similarly, after 15 or 30 days of storage, the decay indexes were significantly higher in organic fruit than the conventional fruit.

### 3.2. Microbial Composition

**Figure 3:**
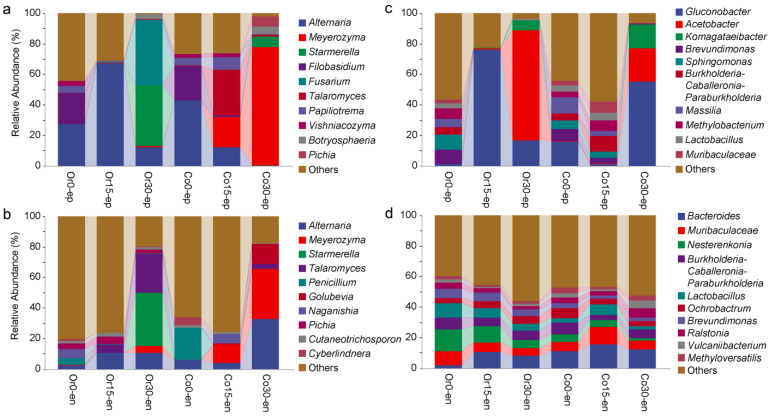
Microbial composition at the genus level in ‘Huangguan’ pears from organic and conventional orchards. (**a**): epiphytic fungi. (**b**): endophytic fungi. (**c**): epiphytic bacteria. (**d**): endophytic bacteria. Five replicates were used in the microbial analysis.

To clarify the main taxa of microorganisms in fruit from different pest management systems, the resulting composition of the 10 most abundant fungal and bacterial communities were analyzed at the genus level (Figure 3).

For the epiphytic fungi, at the beginning of storage, the dominant genera were *Alternaria* and *Filobasidium*, in both organic (Or0, 20.65%) and conventional (Co0, 22.64%) fruit. However, the abundance of *Filobasidium* decreased rapidly to less than 1% after 15 and 30 days of storage (Figure 3a). The *Alternaria* increased from 27.26% to 67.20% in organic fruit after 15 days of storage, followed by a decrease to 12.20% after 30 days. It was found to be gradually decreased on day 0 (42.85%), 15 (11.97%) and 30 (0.28%) of storage in conventional fruit. Compared with day 0 of storage, *Meyerozyma* (19.93%) and *Talaromyces* (30.31%) were higher in the conventional fruit after 15 days of storage compared to day 0. After 30 days of storage, *Fusarium* (43.18%) and *Starmerella* (39.81%) were the dominant fungi in organic fruit while *Meyerozyma* (77.74%) was the dominant fungus in the conventional fruit.

For the endophytic fungi, the microbial composition was different compared to the epiphytic fungi (Figure 3b). *Penicillium* was present in both organic (3.78%) and conventional fruit (20.53%) at day 0, and then, at the end of storage, decreased to 0.55% and 0% in organic and conventional fruit, respectively. After 30 days of storage, *Talaromyces* (25.46%) and *Starmerella* (34.89%) were the dominant fungi in organic fruit, while *Meyerozyma* (32.80%) and *Golubevia* (13.15%) were the dominant fungi in conventional fruit.

For the epiphytic bacteria, *Gluconobacter* (76.23%) was dominant in organic fruit after 15 days of storage (Figure 3c). After 30 days of storage, *Acetobacter* (71.81%) and *Gluconobacter* (16.75%) were more abundant in organic fruit, while no obvious change was observed in the complex dominant microbial community at 15 days in conventional fruit. *Acetobacter* (21.60%) and *Gluconobacter* (55.05%) were more abundant at day 30 in the conventional fruit. For the endophytic bacteria, their composition and structure in the organic and conventional fruit were relatively similar during storage. The *Bacteroides*, *Muribaculaceae*, and *Nesterenkonia* were the main dominant bacteria observed in both fruit (Figure 3d).

### 3.3. Alpha Diversity Analysis

**Figure 4:**
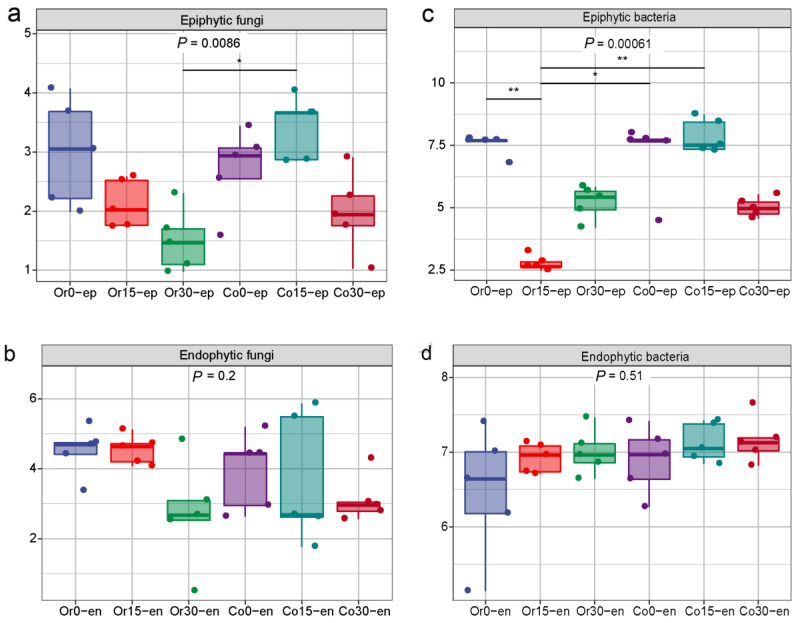
Alpha diversity analysis of microorganisms in ‘Huangguan’ pears from organic and conventional orchards. (**a**): epiphytic fungi. (**b**): endophytic fungi. (**c**): epiphytic bacteria. (**d**): endophytic bacteria. The *p*-value of the Kruskal-Wallis test is displayed, and the Dunn’s test for post-hoc analysis is indicated with an asterisk (*) to mark significance.

For the epiphytic fungi, Shannon index gradually decreased with time in both organic and conventional fruit, indicating a decrease in species richness (Figure 4a). However, Shannon index decreased more rapidly in organic fruit, implying that dominant fungi appeared. For the endophytic fungi in the organic and conventional fruits, no significant change was shown during storage, which indicates that the endophytic fungi richness was stable (Figure 4b). For the epiphytic bacteria, the massive enrichment of *Gluconobacter* in organic fruit after 15 days led to a rapid decrease in the Shannon index, and the appearance of other bacteria such as *Acetobacter.* The appearance of *Acetobacter* after 30 days led to an increase in the microbial richness (Figure 4c). No significant change was observed in the endophytic microorganisms during the storage period in both fruits (Figure 4d).

### 3.4. Beta Diversity Analysis

**Figure 5:**
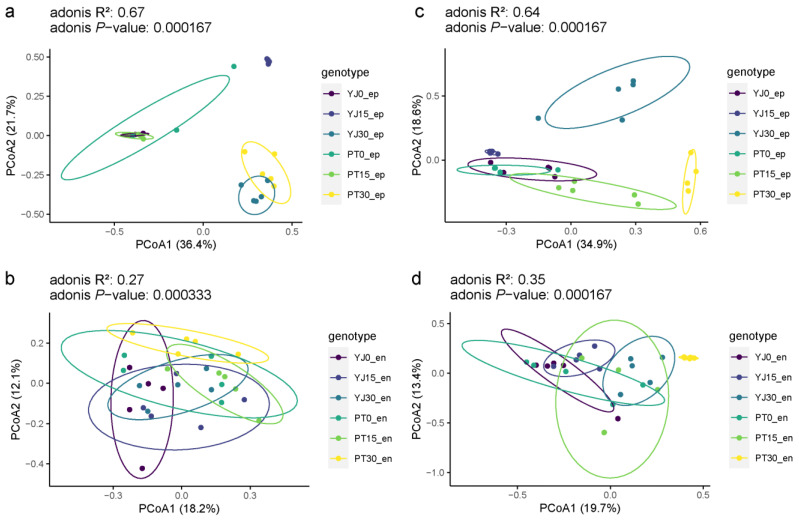
PCoA analysis of microorganisms in ‘Huangguan’ pears from organic and conventional orchards. (**a**): epiphytic fungi. (**b**): endophytic fungi. (**c**): epiphytic bacteria. (**d**): endophytic bacteria.

PCoA was used to employ Bray–Curtis calculations at a confidence level of 0.95 (Figure 5, Table S1). For the epiphytic fungi, microbial diversity in organic and conventional fruits were significantly different (*p* = 0.0096) after 15 and 30 days of storage, while no significant difference (*p* = 0.4522) was found at the beginning of storage. This suggests as storage progressed, the structure of the epiphytic microbial community changed, and was significantly different between fruit from different orchards (Figure 5a).

For the endophytic fungi, although there were significant differences between the organic and conventional fruits after 15 (*p* = 0.0453) and 30 (*p* = 0.0203) days of storage, the differences were smaller compared to those of the epiphytic fungi (Figure 5b).

The epiphytic bacteria microbial diversity in organic fruits were significantly different from those in conventional fruits after 15 and 30 days of storage (Figure 5c). For the endophytic bacteria, no significant difference (*p* = 0.2827) was observed between the two fruits after 15 days of storage, while a significant difference (*p* = 0.0260) was observed after 30 days of storage (Figure 5d).

### 3.5. Random Forest Analysis for Microbial Biomarkers of Organic and Conventional Pear Fruit

**Figure 6:**
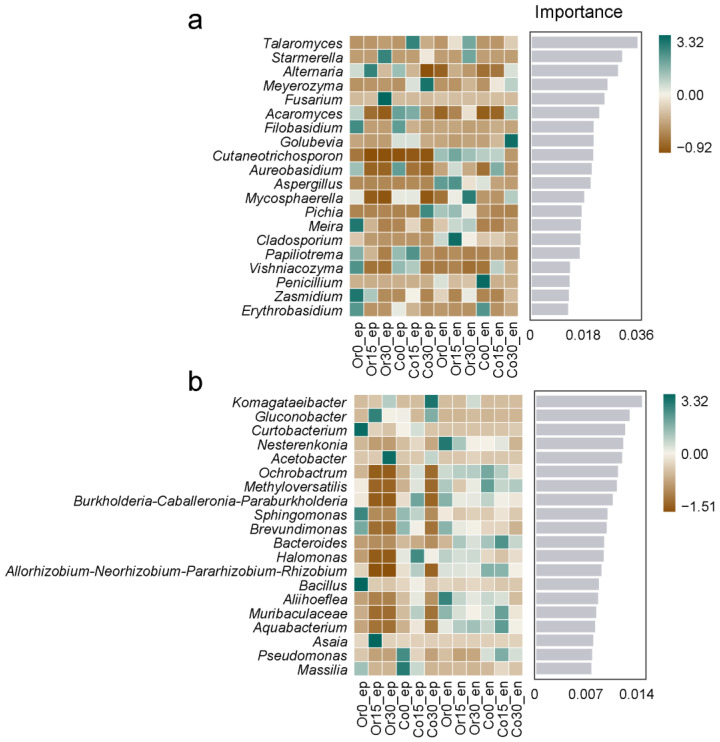
Differences in random forest analysis for fungi (**a**) and bacteria (**b**) in ‘Huangguan’ pears from organic and conventional orchards. The horizontal coordinates represent the important values for the classifier model and the vertical coordinates represent the taxonomic unit names at the genus level, which can be considered as microbial biomarkers for each corresponding treatment.

Random forest analysis was used to show the unique species in different fruits [29]. The top 20 most important genera are listed in Figure 6, and their abundance distribution was plotted as a heat map. *Fusarium* and *Starmerella* were identified as the epiphytic fungal markers for the organic fruit, which had severe decay after 30 days of storage, while *Meyerozyma* was the marker for the conventional fruit. After 30 days of storage, *Mycosphaerella* can be regarded as the endophytic fungal marker for the organic fruit while *Golubevia* was the endophytic fungal marker for the conventional fruit. For bacteria, *Acetobacter* was identified as the epiphytic marker after 30 days of storage for the organic fruit, while *Komagataeibacter* was the epiphytic marker for the conventional fruit.

### 3.6. Relationship between Fruit Quality, Decay, and Microbial Composition in ‘Huangguan’ Pear Fruit

**Figure 7:**
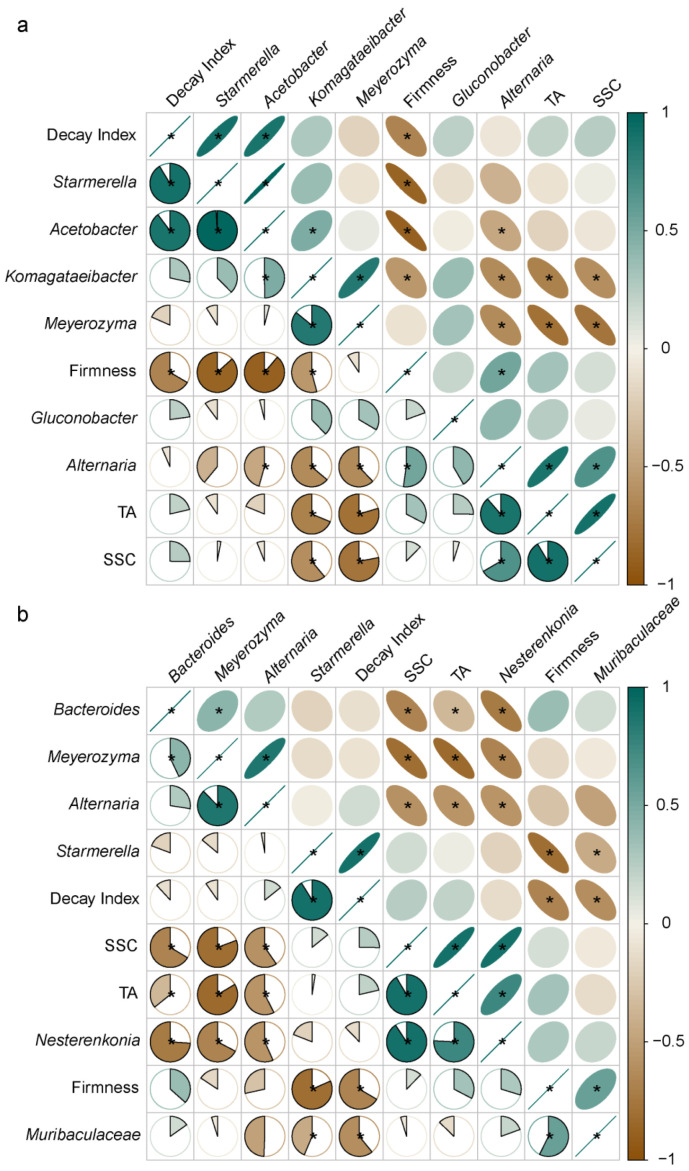
Relationship between fruit decay, quality, and microbial composition in ‘Huangguan’ pear fruit. (**a**), epiphytic microorganisms. (**b**), endophytic microorganisms. * represents significance at *p* < 0.05 level.

In order to study the relationship between fruit quality, decay, and major microorganisms, we selected the three epiphytic and endophytic microorganisms with the highest abundance, and analyzed their correlation to fruit quality. Results from Figure 7 show that decay index was negatively correlated with fruit firmness, suggesting that fruit softening may facilitate pathogen invasion. The abundance of *Acetobacter* in the epiphytic area, and *Starmerella* in both the epiphytic and endophytic area, were found to have a positive correlation with fruit decay, while the abundance of endophytic *Muribaculaceae* was negatively correlated with fruit decay. In addition, the fruit firmness was negatively correlated with the abundance of three epiphytic microorganisms including *Komagataeibacter*, *Acetobacter*, and *Starmerella*, while endophytic *Muribaculaceae* was positively correlated with fruit firmness. These results suggest that epiphytic *Acetobacter* and *Starmerella* may be involved in the reduction of fruit firmness and increase of decay, while endophytic *Muribaculaceae* played the opposite role.

Furthermore, SSC was positively correlated with the abundance of epiphytic *Alternaria* but negatively correlated with epiphytic *Meyerozyma* and *Komagataeibacter*, and endophytic *Alternaria*. TA was positively correlated with epiphytic *Alternaria* and endophytic *Nesterenkonia*, while it was negatively correlated with epiphytic *Meyerozyma* and *Komagataeibacter*, and endophytic *Alternaria*, *Meyerozyma*, and *Bacteroides*.

**Figure 8:**
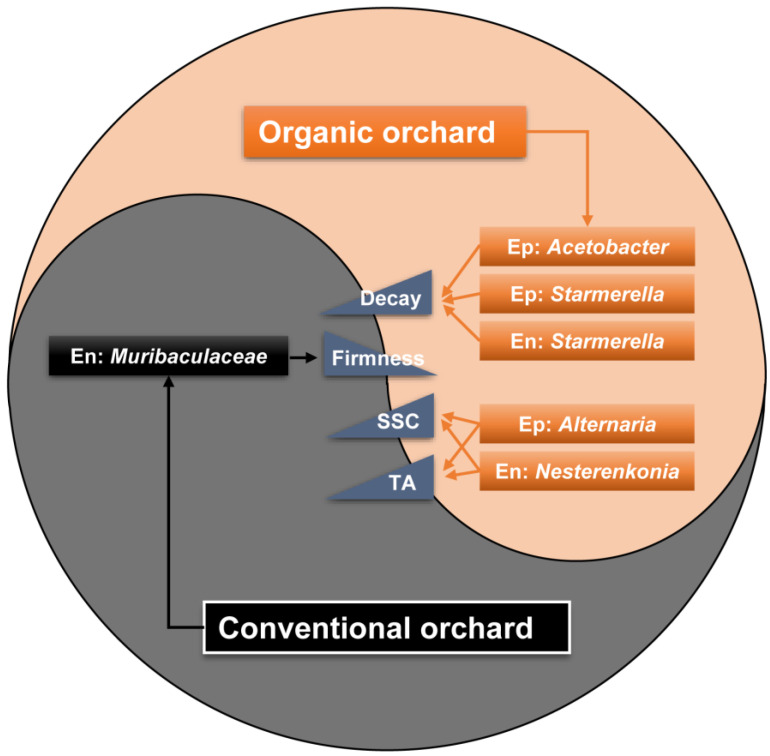
Model proposed for the relationship of microbiome and fruit quality in organic and conventional ‘Huangguan’ pear fruit.

In summary, changes in the microbial structure of pear fruits from organic orchards affect the quality and decay of fruits during postharvest shelf-life storage (Figure 8). The increase of the epiphytic *Acetobacter*, *Starmerella* and endophytic *Starmerella* population may cause fruit decay, which was closely related to loss of firmness. Additionally, the increase in SSC and TA, which resulted from the ripening process, may be associated with the accumulation of epiphytic *Alternaria* and endophytic *Nesterenkonia* in fruit.

## 4. Discussion

Organic fruits are becoming increasingly popular and are gaining a larger share of the market. As people become more aware of the importance of health and environmental protection, the demand for organic fruits is expected to continue to grow. Choosing organic fruits not only benefits our own health but also helps to protect the environment for future generations. Organic fruits are grown in an environmentally friendly manner that has a positive impact on the natural environment and helps to protect and restore biodiversity. The cultivation of organic fruits does not pollute the soil, water sources, or air, and does not disrupt the ecological balance [30]. Instead, it coexists harmoniously with nature. Organic fruits are also healthier than conventionally grown fruits because they do not contain artificial substances, such as pigments, preservatives, and colorants. They are pure natural foods that better meet the physiological needs of the human body. Organic fruits can provide a rich source of vitamins, minerals, antioxidants, and other nutrients that can help to boost our immune system and prevent various diseases [31].

In this study, the effects of organic and conventional orchard management on fruit quality and microbiomes were evaluated in the ‘Huangguan’ pear. During storage, the decay incidence and decay index in organic fruit were significantly higher than in conventional fruit. Orchard management systems have been reported to affect microbial communities in several fruits [22,32]. Organic orchards may be more susceptible to decay during storage because they are managed without the use of pesticides. Pesticides are commonly used in conventional agriculture to protect crops from pests and diseases. However, organic farming practices prohibit the use of synthetic pesticides and instead rely on natural methods to control pests and diseases. While organic farming practices are more environmentally friendly because they do not use synthetic pesticides, this approach may also mean that organic orchards are managed without the application of pesticides. As a result, pathogens may accumulate on the surface of fruits while they are still in the field. This can increase the risk of fruit disease during postharvest storage. Without the protection provided by pesticides, organic fruits may be more susceptible to damage from pests and diseases, which can lead to decay and spoilage [33]. In addition, some microorganisms affect fruit firmness, and changes in their abundance would also affect the decay incidence. However, we acknowledge that fruit firmness is only one aspect of rheological behavior. Other parameters, such as deformation at the breaking point, work to break, and stiffness, may also be relevant for characterizing fruit quality and susceptibility to decay. Moreover, other factors besides microbiota may influence fruit softening, such as ethylene production, cell wall degradation, and water loss. Therefore, further studies with larger sample sizes and more comprehensive rheological measurements are needed to confirm our findings and elucidate the mechanisms underlying fruit senescence and decay.

*Alternaria* and *Fusarium*, which has been reported as two of the most common pathogens and toxin-producing fungi, were highly prevalent in the organic fruit during storage [34,35,36,37]. It has been shown that *Alternaria*, a typical latent infectious fungus, can infect fruits and cause postharvest decay in apples, pears, and pomegranates, etc. [38,39,40]. This major pathogen causes Alternaria rot in pear fruit by infecting the young fruit during growth and remaining latent until the fruit matures [41]. *Fusarium* is a virulent species, pathogenic to plants and humans and capable of colonizing a wide range of environments [42]. *Fusarium* was associated with fruit disease in banana [4], grape [43], and pear, and it seriously affects fruit quality and yield. These two fungal genera were also identified as markers in organic fruit, indicating that they played important roles in the decay of the ‘Huangguan’ pear.

In the later stages of storage, the abundance of *Starmerella* increased in organically growing pears. Similar findings have been reported for winemaking. This pathogen is able to synthesize various metabolites that may impact the chemical composition of grape juice and wine [44,45]. In contrast, in pears from conventional orchards, although the content of pathogenic fungi was higher in the pre-storage period, it decreased in the mid and late-storage periods. *Talaromyces* and *Meyerozyma* were identified to be dominant after 15 days of storage in conventional fruit. *Talaromyces* can produce secondary metabolites that inhibit spore germination and mycelial elongation of *Fusarium* [46]. *Meyerozyma* is an uncommon ascomycete yeast that can utilize various carbon sources and has been widely applied in industrial enzyme production, metabolite synthesis, and biocontrol. In particular, it can increase the antioxidant enzyme activity in pears, thus enhancing disease resistance [47,48]. The high load of *Meyerozyma*, a marker species in conventional pear fruit at day 30 of storage, suggests that this microorganism may play a positive role in the control of fruit decay. The pathogens detected initially (i.e., at harvest; day 0), on the surface of conventional pear fruit, were replaced by yeast after 15 days of storage. This may be the reason why the incidence of decay was lower in conventional fruit than in organic fruit.

Previous reports have shown that orchard management systems and cultivation methods can affect fruit quality [49,50,51]. In this present work, the result showed that compared to conventionally grown pears, organic fruit maintained higher SSC and TA during storage, but was softer after 30 days of storage. A considerable number of bacterial species have been found to be beneficial for plant growth and contribute to fruit quality. For instance, floral and foliar applications of *Bacillus* sp. (plant growth promoting bacteria) on cherries significantly increased the yield and improved fruit quality [52]. It has been reported that the inoculation of strawberry with *Pseudomonas fluorescens* strain Pf4 alone increased malic acid content in fruits, while decreasing the pH value, and furthermore, it increased the sucrose and sugar content when used together with *Funneliformis mosseae* [53].

Bacterial composition of pear fruit was significantly different between organic and conventional fruits. After storage, the dominant epiphytic bacteria were *Acetobacter* and *Gluconobacter* after 30 days of storage in organic and conventional fruit. *Gluconobacter* strains have been reported to play a role in postharvest losses of fruit by causing rot and browning. They have been isolated repeatedly from rotting apples and pears [54]. Although bacteria in the *Gluconobacter* genus have been reported to have antagonistic effects on fruit fungal diseases, most studies have shown that it can promote fruit rot and cause postharvest loss. *Acetobacter* is a genus of acetic acid bacteria characterized by the ability to convert ethanol to acetic acid in the presence of oxygen. *Acetobacter* is frequently isolated in fruits as a natural strain and can be used for the manufacture of acetic acid in the industry [55]. *Acetobacter* is also known to be a causal agent of bacterial rot in pears and apples, resulting in different shades of browning and tissue degradation [54]. *Acetobacter* may cause both considerable economic profits and losses. The latter aspect results from the spoiling activity in many products that provide sufficient conditions for growth. The differences observed in microbial composition were possibly responsible for the variation in fruit quality, such as in SSC and titratable acidity. However, further experiments are needed to confirm this hypothesis. These experiments could include additional harvests and larger sample sizes to provide more robust data and support for the results of the initial experiment. Additionally, key microorganisms could be isolated and studied to better understand their biological functions and their role in the postharvest storage of pear fruit. By conducting these further experiments, researchers can gain a deeper understanding of the relationship between microbial composition and fruit quality.

## 5. Conclusions

‘Huangguang’ pear fruit grown under different pest management systems, organic and conventional, showed significant differences in firmness, SSC, TA and decay throughout storage. Overall, pears from the organic orchard were more susceptible to decay than fruit from conventional orchards. In both conventional and organic fruits, the endophytic microbial community structure during storage was more stable than that of the epiphytic microbial community. The decay index had a negative correlation with both fruit firmness and the abundance of endophytic *Muribaculaceae*. However, it was positively correlated with the abundance of epiphytic *Acetobacter* and both epiphytic and endophytic *Starmerella*. These results provide a basis for further studies focused on the relationship between microorganisms and fruit decay and quality. Therefore, future work may involve isolating and characterizing epiphytic and endophytic microorganisms from larger samples and multiple harvests to better understand the difference between the two orchard managements.

## Data Availability

The data presented in this study are available on request from the corresponding author. Data of ITS and 16S amplicon sequencing of the samples were submitted to NCBI under the Bioproject Id PRJNA902155.

## Supplementary Materials

The following supporting information can be downloaded at: https://www.mdpi.com/article/10.3390/foods12101980/s1, Table S1: PERMANOVA (adonis) test for the differences of microorganisms between organic and conventional orchards in ‘Huangguan’ pears.
