# Supplementary material for: The Microbial Diversity in Relation to Postharvest Quality and Decay: Organic vs. Conventional Pear Fruit
Source: Foods. 2023 May 12;12(10):1980. doi: 10.3390/foods12101980 (PMC10217483; doi:10.3390/foods12101980)
Supplement: Supplementary file 1 [file foods-12-01980-s001.zip › foods-2266205-supplementary.pdf]

Table S1 PERMANOVA (adonis) test for the differences of microorganisms between organic and conventional orchards in 'Huangguan' pears.

pairs	R2	p.adjusted	significance
**epiphytic fungi**			
Or0_ep vs Or15_ep	0.3581	0.0096	*
Or0_ep vs Or30_ep	0.3959	0.0096	*
Or0_ep vs Co0_ep	0.1147	0.4522	
Or0_ep vs Co15_ep	0.2712	0.0096	*
Or0_ep vs Co30_ep	0.5913	0.0096	*
Or15_ep vs Or30_ep	0.6064	0.0096	*
Or15_ep vs Co0_ep	0.4891	0.0096	*
Or15_ep vs Co15_ep	0.6308	0.0096	*
Or15_ep vs Co30_ep	0.8850	0.0096	*
Or30_ep vs Co0_ep	0.4923	0.0096	*
Or30_ep vs Co15_ep	0.4635	0.0096	*
Or30_ep vs Co30_ep	0.5823	0.0096	*
Co0_ep vs Co15_ep	0.3673	0.0096	*
Co0_ep vs Co30_ep	0.7358	0.0096	*
Co15_ep vs Co30_ep	0.5321	0.0096	*
**endophytic fungi**			
Or0_en vs Or15_en	0.0836	0.8457	
Or0_en vs Or30_en	0.2597	0.0203	*
Or0_en vs Co0_en	0.0885	0.6704	
Or0_en vs Co15_en	0.1611	0.1109	
Or0_en vs Co30_en	0.4412	0.0203	*
Or15_en vs Or30_en	0.2216	0.0203	*
Or15_en vs Co0_en	0.1340	0.3521	
Or15_en vs Co15_en	0.1937	0.0453	*
Or15_en vs Co30_en	0.4100	0.0203	*

Or30_en vs Co0_en	0.2626	0.0203	*
Or30_en vs Co15_en	0.2250	0.0272	*
Or30_en vs Co30_en	0.3855	0.0203	*
Co0_en vs Co15_en	0.1703	0.1210	
Co0_en vs Co30_en	0.4154	0.0203	*
Co15_en vs Co30_en	0.3251	0.0203	*
**epiphytic bacteria**			
Or0_ep vs Or15_ep	0.7483	0.0133	*
Or0_ep vs Or30_ep	0.5688	0.0127	*
Or0_ep vs Co0_ep	0.1316	0.2185	
Or0_ep vs Co15_ep	0.2412	0.0133	*
Or0_ep vs Co30_ep	0.6342	0.0127	*
Or15_ep vs Or30_ep	0.7264	0.0127	*
Or15_ep vs Co0_ep	0.5590	0.0127	*
Or15_ep vs Co15_ep	0.7739	0.0127	*
Or15_ep vs Co30_ep	0.7948	0.0127	*
Or30_ep vs Co0_ep	0.4636	0.0127	*
Or30_ep vs Co15_ep	0.5861	0.0127	*
Or30_ep vs Co30_ep	0.4649	0.0127	*
Co0_ep vs Co15_ep	0.1695	0.0171	*
Co0_ep vs Co30_ep	0.4972	0.0127	*
Co15_ep vs Co30_ep	0.6534	0.0127	*
**endophytic bacteria**			
Or0_en vs Or15_en	0.1566	0.1792	
O0_en vs Or30_en	0.2038	0.0260	*
Or0_en vs Co0_en	0.1934	0.0417	*
Or0_en vs Co15_en	0.2451	0.0260	*
Or0_en vs Co30_en	0.2668	0.0260	*
Or15_en vs Or30_en	0.1384	0.1871	
Or15_en vs Co0_en	0.1045	0.4895	

Or15_en vs Co15_en	0.1253	0.2827	
Or15_en vs Co30_en	0.2112	0.0260	*
Or30_en vs Co0_en	0.1473	0.1871	
Or30_en vs Co15_en	0.1773	0.0600	
Or30_en vs Co30_en	0.2299	0.0260	*
Co0_en vs Co15_en	0.1453	0.1919	
Co0_en vs Co30_en	0.1556	0.1871	
Co15_en vs Co30_en	0.1820	0.0536	
